# Risk factors for tuberculosis in dialysis patients: a prospective multi-center clinical trial

**DOI:** 10.1186/1471-2369-10-36

**Published:** 2009-11-07

**Authors:** Antonios I Christopoulos, Athanasios A Diamantopoulos, Panagiotis A Dimopoulos, Demetrios S Goumenos, George A Barbalias

**Affiliations:** 1 School of Health & Wellfare Professions - Department of Nursing, Higher Technological-Educational Institute, Patra, Greece; 2 Department of Nephrology of "St. Andrew" General State Hospital, Patra, Greece; 3 Department of Radiology, University Hospital Rio, Patra, Greece; 4 Departent of Nephrology, University Hospital Rio, Patra, Greece; 5 Department of Urology, University Hospital Rio, Patra, Greece

## Abstract

**Background:**

Profound alterations in immune responses associated with uraemia and exacerbated by dialysis increase the risk of developing active tuberculosis (TB) in chronic haemodialysis patients (HDPs). In the current study, was determined the impact of various risk factors on TB development. Our aim was to identify which HDPs need anti-TB preventive therapy.

**Methods:**

Prospective study of 272 HDPs admitted, through a 36-month period, to our institutions. Specific Relative Risk (RR) for TB was estimated, considering age matched subjects from the general population as reference group. Entering the study all patients were tested with tuberculin (TST). Using Cox's proportional hazard model the independent effect of various risk factors associated with TB development was estimated.

**Results:**

History of TB, dialysis efficiency, use of Vitamin D supplements, serum albumin and zinc levels were not proved to influence significantly the risk for TB, in contrast to: advanced age (>65 years), BMI, diabetes mellitus, tuberculin reactivity, healed TB lesions on chest X-ray and time on dialysis. Elderly (>70 years old) HDPs (Adjusted RR 25.3, 95%CI 20.4-28.4, P < 0.02), diabetics (Adj.RR 25.3, 95%CI 17.2-21.1, P < 0.03), underweighted (Adj.RR 72.3, 95%CI 65.2-79.8 P < 0.001), tuberculin responders (Adj.RR 41.4, 95%CI 37.9-44.8, P < 0.03), HDPs with fibrotic lesions on chest x-ray (Adj.RR 82.3, 95%CI 51.3-95.5, P < 0.03) and those treated with haemodialysis for < 12 months (Adj.RR 110.0, 95%CI 97.4-135.3, P < 0.001), presented significantly higher specific RR for TB even after adjusting for the effect of the remaining studied risk factors.

**Conclusion:**

The above mentioned factors have to be considered by the clinicians, evaluating for TB in HDPs. Positive TST, the existence of predisposing risk factors and/or old TB lesions on chest X-ray, will guide the diagnosis of latent TB infection and the selection of those HDPs who need preventive chemoprophylaxis.

## Background

Profound alterations in immune responses associated with uraemia and exacerbated by dialysis, increase the risk for developing active tuberculosis (TB) after primary infection [1]. Frequent hospital contacts, older age and use of immunosuppressive drugs are additional factors explaining the higher prevalence of TB in these patients.

Tuberculosis in end-stage renal disease patients on regular haemodialysis (HDPs) presents a number of diagnostic challenges. In these patients the symptomatology of TB is often insidious and non specific, whereas the localization is often extrapulmonary [2,3]. This makes the disease difficult to diagnose, delaying the initiation of curative treatment, which is a major determinant of the outcome [2-4]. That is why annual skin testing with tuberculin skin test (TST) and chemoprophylaxis for all HDPs with a ≥10 mm response is recommended [5]. However, even today (with increased dialysis quality and dose) there is a high rate of anergy reported among HDPs and anergic HDPs are at increased risk of developing TB [6].

The aim of our study was to identify those HDPs who would benefit from TB chemoprophylaxis, using a combine approach of risk factors and common screening methods, like TST and chest X-ray. Specific-risks for TB were estimated as accordingly to TST results and chest X-ray findings, as well as history of TB, clinical important determinants, medication and specific dialysis related factors like dialysis efficiency and time on dialysis. Associating the later with the risk for TB we were also able to identify when the appropriate time to commence TB chemoprophylaxis in HDPs is.

## Methods

### Study design

This was a prospective, multi-centre trial conducted over the course of three years in two chronic haemodialysis outpatient units affiliated with the University Hospital of Patras and a third unit in the Nephrology Department of the General State Hospital in Patras. The study protocol was approved by the ethical committee of the medical school of the University of Patras. Patients files were searched for factors, that might have influenced the risk of developing TB, like: age, primary renal disease, TB history (disease, contact, vaccination), time on dialysis (time elapsed since undergoing haemodialysis), medication (vitamin D supplements and immunosuppressive drugs like corticosteroids, cyclophosphamide, calcineurin inhibitors, azathioprine and mycophenolate mofetil), and laboratory data like serum albumin and zinc levels. Dialysis efficiency was measured using equilibrated dialysis dose (Kt/V) and urea reduction ratio (URR). Body Mass Index (BMI) and serum albumin, were used as markers of the patients' nutritional status.

Entering the study, participants were evaluated, and active TB was ruled out by history, physical examination, chest radiography, and when indicated, bacteriological studies. Following patients were tested with tuberculin. TB incidence and the subsequent relative risk (RR) for TB was then determined over a 36 month follow-up.

All efforts were made for bacteriological and/or histological confirmation. In patients with clinical and/or radiographic findings suggestive of TB, respiratory samples consistent of three sputum specimens (or three gastric washings in the absence of of productive cough) were stained with Ziehl-Neelsen stain and cultured in Lowenstein-Jensen medium for *M. tuberculosis*. A Bronchoalveolaren lavage was also performed for each patient with negative cultures, but highly suggestive features of TB. HDPs with Lymphadenitis had superficial or deep lymph node biopsies. In case of no lymph node extrapulmonary involvement, TB was identified on the basis of clinical findings and positive biopsies and cultures from one or more clinical specimens.

To identify the RR for TB in haemodialysis patients accurately, on a population basis, the Prefecture's Health Service registries, where all TB cases diagnosed are mandatory reported, were also reviewed. From these, the rate of TB, for the age-matched control group was estimated. Participants provided informed consent.

### Participants

Of the 272 end stage renal disease patients who were included in the study, 193 were male and 79 female. Their median age was 52.7 years, ranging from 25 to 77 years. They were treated with dialysis for a mean of 3.9 years (range 1 week to 18.4 years). Primary renal disease was classified in eight categories: glomerulonephritis (48), nephroangiosclerosis (46), diabetic nephropathy (75), polycystic kidneys (22), interstitial nephritis-reflux nephropathy or nephritis due to analgesic abuse (42), infection (15), renal carcinoma (2) and unknown origin (22). Five patients had a history of active pulmonary TB, but all had been treated adequately. Close, non-hospital contacts of persons with infectious TB were reported in seven patients. As Greece is among the countries where BCG vaccination is still in use, most patients have been vaccinated at the age of six years. However, only 173 patients remembered or had scars of vaccination with BCG. Patients on corticosteroids or other immunosuppressive therapy were not included in this study because of the rather small number among the patients dialysed in our units. We did not perform a routine assessment for HIV infection, as in our area prevalence of HIV associated TB is insignificant.

### Tuberculin Skin Testing (TST)

TST was carried out by the intradermal (Mantoux) method. It was administered by injecting 0.1 ml of commercially available tuberculin (PPD-Merieux, 5 U/0.1 ml dose), intradermally into the anterior surface of the forearm, and was read 48-72 hours later. A positive TST was a skin induration ≥10 mm (5-9 mm weak-positive and 0-4 mm negative). A second booster injection with 10 U/0.1 ml dose, was given within a 10-day interval to those HDPs responding with a <10 mm induration to the first test. The pen method was used for the measurement of skin tuberculin reaction as previously described [7,8].

### Chest Radiography

Entering the study, all patients had a chest X-ray, evaluated by two independent physicians, (radiologist and pulmonologist). Radiographic findings that could be attributed to previous healed TB were described as: 1) Dense pulmonary nodules, with (group-A, n = 51) or without visible calcification (group-B, n = 36), in the hilar area or upper lobes, 2) smaller nodules with or without fibrotic scars in the upper lobes, accompanied with upper-lobe volume loss (group-C, n = 32) and 3) bronchiectasis of the upper lobe and/or pleural scarring (group-D, n = 37). Nodules and fibrotic lesions of previous, healed TB were prescribed as hard-well-demarcated with sharp margins.

### Statistical methods

TB prevalence was defined as the number of TB cases per a hypothetical population at risk of 100. Analysis of variance (*ANOVA*) was used to assess the statistical significance of differences. For multivariate analysis, only factors that were significantly different (P < 0.05) in univariate comparison were retained. The independent effects of the significant factors, was determined using a multivariate analytical method the *Cox's *proportional hazard model. [9] Trends in prevalence and risk for TB through the different studied factors were assessed by *Spearmans's *product moment correlation coefficient (r), a dimensionless index that ranges from -1.0 to 1.0, reflecting the extent of the relationship between data sets.

## Results

### Findings of the tuberculin skin testing

Upon entering the study, the prevalence of a positive TST was 18.9% for the first and 22.7% (n = 62) for the second test. Twenty-seven patients (9.9%) demonstrated a weak-positive response. A negative response to TST was recorded in 183 HDPs (67.2%). (Table 1)

**Table 1:** The risk for TB in dialysis patients by TST at onset, chest radiography and dialysis stage

*Characteristic (%)*	*Population at risk*	*TB cases*	*TB rate*	*RR*	*Adj RR*	*95% CI*
**TST at inclusion (mm)**						
0-4	183	14	7.65	69.5	24.5	22.5-26.5
5-9	27	2	7.40	67.2	8.4	3.1-13.6
≥10	62	8	12.0	109.0	41.4	37.9-44.8
*r*			*0.84*		*0.51*	
*P value*			*0.02*		*0.03*	
**Inactive-TB evidence on chest x-ray**						
Yes	67	10	14.9	135.4	82.3	51.3-95.5
No	205	14	5.4	49.9	21.3	19.8-22.8
*Ratio*	*0.06*		*10.8*	*2.7*	*3.8*	
*P value*			* < 0.001*		* < 0.001*	
**Dialysis stage in months**						
0-12	67	16	23.82	216.3	110.0	97.4-135.3
13-60	149	3	2.01	18.3	9.3	5.2-14.6
≥60	56	5	8.92	81.0	41.3	31.3-56.8
*r*			*-0.46*		*-0.58*	

*P value*			* < 0.001*		* < 0.001*	

**RR**: relative risk taking as reference group subjects from the general population, **Adj RR**: adjusted relative risk after allowing for the effect of the other characteristics, **CI**: Wald 95% Confidence Interval, **r**: correlation coefficient, **P value**: for the differences between groups

### TB incidence

During the 36 month follow up period, 24 HDPs developed active TB, a rate of 8.2%. TB incidence was significantly higher in diabetics (P = 0.01), increased with increasing age *(r = 0.95, P = 0.006) *and tuberculin reactivity at inclusion *(r = 0.84, P = 0.02)*, while decreased with increasing BMI *(r = -0.98, P < 0.001)*. (Tables 1 and 2) Seventeen of the HDPs with negative TSTs at inclusion (17/183) developed active TB. Thirteen of them (13/17) had negative TSTs even when the active disease developed.

**Table 2:** The risk for TB in dialysis patients, by sex, age, Body Mass Index (BMI) and Diabetes Mellitus.

*Characteristic*	*Population at risk*	*TB cases*	*TB rate (%)*	*RR*	*Adj RR*	*95% CI*
**Sex**						
Male	193	15	7.7	70	28.2	26.4-30.1
Female	79	9	11.3	102.7	34.5	31.4-37.5
*M:F ratio*	*2.4*		*0.6*		*0.8*	
*P value*			*0.003*		*0.01*	
**Age**						
≤ 49	38	2	5.2	47.8	16.2	11.7-20.6
50-69	165	12	7.2	66.1	22.8	20.6-24.9
≥ 70	69	10	14.4	130.9	25.3	20.4-28.4
*r*			*0.95*		*0.95*	
*P value*			*0.006*		*0.02*	
**BMI**						
< 18	58	9	15.5	140.9	72.3	65.2-79.8
18-25	79	7	8.8	80.0	36.8	28.3-44.5
25-30	92	6	6.5	59.0	28.4	22.1-34.3
>30	43	2	4.6	42.2	23.1	18.4-28.2
*r*			*-0.98*		*-0.99*	
*P value*			* < 0.001*		* < 0.001*	
Diabetes Mellitus						
Yes	75	8	10.6	96.3	25.3	20.4-28.4
No	197	16	8.1	73.6	19.2	17.2-21.1
*DM:no DM ratio*	*0.3*		*1.3*		*1.3*	
*P value*			*0.01*		*0.03*	

**RR**: relative risk taking as reference group subjects from the general population, **Adj RR**: adjusted relative risk after allowing for the effect of the other characteristics, **CI**: Wald 95% Confidence Interval, **r**: correlation coefficient, **P value**: for the differences between groups.

In 4 of the cases (16.6%), lungs were the only site of involvement. Extra-pulmonary manifestations were verified in the remaining 20 cases (83.4%), with lymph nodes, pleural, pericardium, peritoneum, liver, spleen, kidneys and bones being the sites of involvement reported.

### Association of chest X-ray with TB incidence

From the 67 patients with findings suggestive of old-healed TB in chest radiographs, upon entering the study, 10 (10/67, 14.9%) developed active TB. Three of them, with group-B, six with group-C and one with group-D type of old-healed TB lesions in the chest radiographs. The rate of TB was: 0, 8.3, 18.7 and 2.7% for A, B, C and D group respectively. Totally, HDP with old-healed TB lesions on chest radiographs demonstrated an almost three-fold higher risk for developing active TB as compared to HDPs without such lesions (14.9% *vs *5.4%). (Table 1)

Lesions were described in: 27 (27/62, 43.5%) patients with positive, 8 patients (8/27, 29.6%) with weak-positive and 32 patients (32/183, 17.4%) with negative TST). Using both TST and chest radiography 102 HDP (37.5%) were identified as having been infected with M. Tuberculosis.

### Findings of the univariate analysis

The demographic and other clinical important determinants of those HDPs who developed active TB are presented in Table 3, in comparison with the characteristics of those HDP who did not develop TB during the period of the follow up. The univariate comparison of non TB *vs. *TB HDPs, showed no significant differences as to: efficiency of dialysis Kt/V (P = 0.30), URR (P = 0.97), serum levels of albumin (P = 0.98), serum levels of zinc (0.27), use of vitamin D supplements (P = 0.66) and BCG vaccination (P = 0.92). There were no cases of TB among HDPs with previous history for TB (disease or contact). During the same period, 131 cases of TB were reported to the Public Health Services' registry of Achaia. The prevalence of TB for the age-matched general population was 0.11%.

**Table 3:** Comparison of the baseline characteristics of the dialysis patients who developed/or not TB.

Characteristic	non TB Dps	TB Dps	P values
**Number**	248	24	
Mean age (years)	50.8 (27-75)	61.5 (16-77)	0.03
Sex (male: female ratio)	2.5	0.6	< 0.001
Time on dialysis (years)	3.9 (0.12-18.6)	2.2(0.06-17.8)	0.01
Kt/V	1.49 (1.33-1.67)	1.51 (1.35-1.71)	0.30
URR	71(61-77)	72(61-77)	0.97
Response to tuberculin (mm)	4.21(0-12)	7.16 (0-20)	< 0.001
Body mass index	23 (12-28)	15 (14-32)	< 0.001
Vitamin D (no/yes)	83	10	0.66
Users: no users ratio	0.33	0.4	
Albumin (g/l)	35.2 (33-37.35)	32 (33.2-37)	0.98
Zinc (μg/dl)	77 (69-86)	75 (68.75-83)	0.27
Diabetics (DM)	67	8	0.04
DM: non DM ratio	0.2	0.3	
Old TB evidence on x-Ray	51	16	
abnormal: normal x-Ray ratio	0.2	0.6	< 0.001
Previous TB disease	5	0	
Previous TB contact	7	0	
BCG vaccination	167	15	0.92

Vaccinated: non vaccinated ratio	0.67	0.62	

### Findings of the multivariate analysis

Age, BMI, incidence of diabetic nephropathy, chest X-ray lesions of old healed TB, initial tuberculin sensitivity and time on dialysis were significantly different factors retained for multivariate analysis. (Table 3) The estimated adjusted RRs (Adj.RRs) were appreciably smaller than the respective RRs, due to the high correlation between the studied factors. However, even after allowing for the effect of the other studied factors, the risk for TB remained significantly higher in diabetics (P = 0.03)and those patients with chest radiographs suggestive of old-healed TB (P < 0.001). Furthermore, increasing trends with increasing age *(r = 0.95) *and decreasing BMI (r = 0.99, P < 0.001) were still demonstrated (Tables 1 and 2). The Adj.RR for the tuberculin skin test result of 0-4 mm group was significantly lower in comparison to the respective value for the ≥10 mm group (P < 0.001). However, the Adj.RR for the 5-9 mm group was significantly lower than the respective value for the 0-4 mm group *(P = 0.002)*, resulting finally in a weaker correlation *(r = 0.51) *between tuberculin sensitivity and the risk for developing TB. (Table 1)

Patients undergoing dialysis for a shorter than 12 months period were presented with significantly higher risk for TB, as compared to patients treated for a longer period *(P < 0.001)*. The lowest risk for TB was recorded in patients treated with dialysis for 13 to 60 months. However, a significant re-increase in the risk for TB was observed in patients treated for more than 60 months *(P < 0.001)*. (Table 1)

## Discussion

Worldwide TB infection in dialyzed patients ranges from 5-25% and a 6.9-52.5-fold risk of TB is reported as compared to the general population [[3,10,11], and [12]]. These findings were verified in our study too. The absence of significant differences in Mantoux positivity and/or the incidence of active TB among the three medical centres involved in our study and the high incidence of extra pulmonary forms minimized the impact of clustering and/or the possibility of nosocomial spread.

Advanced age, diabetes mellitus and low BMI, are known factors of immunological imbalance, associated with TB development in the general population. In HDPs they have been also associated with depressed cell-mediated immune responses and anergy [6,13-17]. In our study, they were proved to be factors predisposing to active TB development in haemodialysis patients too. That is why they have to be considered evaluating for TB in the dialysis settings. Their existence supports the decision to prevent or even to treat when definitive diagnosis of clinical manifested TB is not possible, although there is strong presumptive evidence.

Most authors agree that to provide substantial protection against TB in HDPs, chemoprophylaxis should be commenced in all patients with a positive TST [5]. In the general population, TST remains the most satisfactory tool for diagnosing TB infection, and initial tuberculin sensitivity was well associated with the incidence of TB [18]. Boosted TSTs are usually recommended for immunocompromised patients like HDPs [19,27]. Most authors agree that in the hemodialysis population two consecutive tests (within to a 7-14 days interval) with a higher strength (usually double) purified protein derivative, are sufficient to rule out the booster effect, maximizing the identification of the infected patients [20-22]. However, in the current study, HDPs presented a weaker association between initial tuberculin sensitivity and the subsequent risk for TB. A substantial proportion of the HDPs that developed TB, had negative TSTs upon entering the study (14/24) and/or when the active disease was developed (8/24). As anergy distorted tuberculin reactivity, TST underestimated the incidence of TB infection among our patients [6,23].

Unlike the TST, measurement of PPD reactivity by in vitro quantification of PPD-specific T cells has been reported unaffected by uremia-associated immunosuppression [24]. Nowadays, using a rapid 6-hour assay we can enumerate peripheral blood-derived interferon-γ secreting T cells responding to epitopes from an early secretory antigenic target-6 (ESAT-6) that is highly specific for M. *Tuberculosis *complex but absent from M. *Bovis *bacillus Calmete-Guerin (BCG) vaccine strains. Such whole-blood assays may thus be valuable alternative to skin testing in no endemic regions, but they are not still available for the National Health System of Greece [25,26].

Obviously in HDPs, a negative TST has a comparatively low prognostic and diagnostic importance. However, as HDPs with positive TSTs presented increased risk for developing TB, a positive TST should never be underestimated.

Persons with abnormalities on chest radiographs consistent with prior, healed TB have a high risk for progression to active TB (2.0-13.6 per 1000 person-years of observation). Nodules and fibrotic changes may contain slowly multiplying tubercle bacilli with substantial potential for future progression to active TB [27]. HPDs with radiographic evidences of healed TB, demonstrated an almost 4-fold higher risk of developing active TB, independently of the result of the TST, even after allowing for the effect of the remaining risk factors. In our study the radiographic lesions were categorized and the specific significance of their appearance was assessed. Patients with non-calcified nodules and fibrotic changes in the upper lobes of the lungs, presented with the highest risk of TB. Bronchiectasis and pleural scarring are nonspecific findings that sometimes occur from previous pulmonary TB, but are more commonly caused by trauma or other infections. Conversely, calcified nodular lesions (calcified granulomas) and apical or basal pleural thickening posed a lower risk for future progression to active TB. In the case of HDPs such lesions may also represent metastatic calcifications attributed to the secondary hyperparathyroidism, a common clinical situation in uremic patients [28].

The weak correlation between chest radiography and TST positivity recorded in our, as well as in previous studies, verifies the value of chest X-ray in detecting TB infection in haemodialysis patients [29]. Commencing chemoprophylaxis in all HDPs with evidences of healed TB on chest X-rays, especially when diabetic nephropathy is an issue and/or when HDPs are malnourished and they have not been treated adequately in the past, regardless of their response to TST, will improve the control and prevention of TB in this high risk population.

An additional issue, investigated in our study, was the appropriate time for screening with TST and eventually commencing TB chemoprophylaxis in HDPs. There was a significantly higher prevalence and risk for TB recorded, in those patients dialysed for a shorter than a 12 month period. A result well associated with the depressed cell-mediated immune responses recorded in HDPs during an early dialysis stage [6]. It is important to notice that 16 out of the 24 TB cases developed before the first 12 months elapsed since undergoing dialysis, a finding verified in previous studies too [2,30].

In our study, several unresolved issues concerning the identifications of those HDPs who would benefit with TB chemoprophylaxis, were investigated. Calcitriol, which is supposed to restore partly the lymphocyte function and low zinc levels that had been associated with higher anergy [13,14] were not correlated with the development of TB. The efficiency of dialysis didn't influence the risk for TB either.

## Conclusion

The above observations allow us to propose that in order to provide substantial protection against TB in HDPs, the annual skin testing which is recommended, has to be changed to testing upon admission to a regular dialysis program. HDPs with a positive TST and/or lesions suggestive of old healed TB in a recent chest X-ray are candidates for preventive therapy. As diabetic nephropathy and/or low BMI were proved to be independed factors of TB development, their existence supports also the decision to treat. Exception should be made for the elderly HDPs as they are more prone to the pharmacological toxicity of isoniazid [31]. However, as advanced age (> 65 years old) is an independed factor predisposing to TB, the need for a high index of suspicion and timely diagnostic work up should be reinforced in this group of HDPs. Tuberculin skin test and chest radiography upon entering a chronic dialysis program will provide a baseline with which to compare subsequent tests.

## Competing interests

The authors declare that they have no competing interests.

## Authors' contributions

All authors participated in the follow up of the patients and the diagnostic procedure. AC designed the study, performed the statistical analysis and drafted the manuscript. DA participated in the design of the study. DP performed the radiographic evaluation of the patients and helped to draft the paper. GD participated in the design of the study. BG participated in the coordination of the institutions involved and helped to draft the manuscript. All authors read and approved the final version for publication.

## Pre-publication history

The pre-publication history for this paper can be accessed here:

http://www.biomedcentral.com/1471-2369/10/36/prepub
